# Social Media Use and Access to Digital Technology in US Young Adults in 2016

**DOI:** 10.2196/jmir.7303

**Published:** 2017-06-07

**Authors:** Andrea C Villanti, Amanda L Johnson, Vinu Ilakkuvan, Megan A Jacobs, Amanda L Graham, Jessica M Rath

**Affiliations:** ^1^ Schroeder Institute for Tobacco Research and Policy Studies at Truth Initiative Washington, DC United States; ^2^ Department of Psychiatry University of Vermont Burlington, VT United States; ^3^ Department of Health, Behavior & Society Johns Hopkins Bloomberg School of Public Health Baltimore, MD United States; ^4^ Milken Institute School of Public Health Department of Prevention and Community Health George Washington University Washington, DC United States; ^5^ Innovations Truth Initiative Washington, DC United States; ^6^ Department of Oncology Georgetown University Medical Center/Cancer Prevention & Control Program Lombardi Comprehensive Cancer Center Washington, DC United States; ^7^ Evaluation Science and Research Truth Initiative Washington, DC United States; ^8^ Department of Behavioral and Community Health University of Maryland School of Public Health College Park, MD United States

**Keywords:** social media, technology, young adults

## Abstract

**Background:**

In 2015, 90% of US young adults with Internet access used social media. Digital and social media are highly prevalent modalities through which young adults explore identity formation, and by extension, learn and transmit norms about health and risk behaviors during this developmental life stage.

**Objective:**

The purpose of this study was to provide updated estimates of social media use from 2014 to 2016 and correlates of social media use and access to digital technology in data collected from a national sample of US young adults in 2016.

**Methods:**

Young adult participants aged 18-24 years in Wave 7 (October 2014, N=1259) and Wave 9 (February 2016, N=989) of the Truth Initiative Young Adult Cohort Study were asked about use frequency for 11 social media sites and access to digital devices, in addition to sociodemographic characteristics. Regular use was defined as using a given social media site at least weekly. Weighted analyses estimated the prevalence of use of each social media site, overlap between regular use of specific sites, and correlates of using a greater number of social media sites regularly. Bivariate analyses identified sociodemographic correlates of access to specific digital devices.

**Results:**

In 2014, 89.42% (weighted n, 1126/1298) of young adults reported regular use of at least one social media site. This increased to 97.5% (weighted n, 965/989) of young adults in 2016. Among regular users of social media sites in 2016, the top five sites were Tumblr (85.5%), Vine (84.7%), Snapchat (81.7%), Instagram (80.7%), and LinkedIn (78.9%). Respondents reported regularly using an average of 7.6 social media sites, with 85% using 6 or more sites regularly. Overall, 87% of young adults reported access or use of a smartphone with Internet access, 74% a desktop or laptop computer with Internet access, 41% a tablet with Internet access, 29% a smart TV or video game console with Internet access, 11% a cell phone without Internet access, and 3% none of these. Access to all digital devices with Internet was lower in those reporting a lower subjective financial situation; there were also significant differences in access to specific digital devices with Internet by race, ethnicity, and education.

**Conclusions:**

The high mean number of social media sites used regularly and the substantial overlap in use of multiple social media sites reflect the rapidly changing social media environment. Mobile devices are a primary channel for social media, and our study highlights disparities in access to digital technologies with Internet access among US young adults by race/ethnicity, education, and subjective financial status. Findings from this study may guide the development and implementation of future health interventions for young adults delivered via the Internet or social media sites.

## Introduction

Young adulthood, frequently defined as the period from the late teens through the 20s, is a critical time for self-identity construction [1,2]. According to Arnett’s theory of emerging adulthood, identity exploration centers on love, work, and worldviews [2]. Through experimentation with various life possibilities, young people move toward enduring decisions and values during this developmental period. Social media contributes to identity formation by facilitating reflexivity, or the process of reflecting on how we see ourselves and how others see us [3]. In 2015, 90% of US young adults with Internet access used social media [4]. Social media plays an important role in shaping self-beliefs and perceived social norms [5] by inviting other people’s feedback and shaping further modification of self-representations. Social media gives users an opportunity to control their public personae [6] and to experiment with more desirable versions of themselves. This can be done through a number of aspects of social media, including text, images, video, music, affinities (“likes”), visual customization of social media, use of profanity or slang, and group membership or affiliation, to name just a few.

Behavior change theories that address social norms, including social cognitive theory, provide a theoretical framework in which to understand the influence of social media on identity formation [7,8]. Through social observation and interaction, people gather important information from others that can be used to direct their own behavior and beliefs [9] through “electronic acculturation.” Digital and social media are significant modalities through which young adults explore identity formation [10-13], and by extension, learn and transmit norms about health and risk behaviors during this developmental period. Recent studies from a sample of US young adults have also highlighted potentially harmful effects of high social media use on mental health, including lower perceived emotional support [14], greater perceived social isolation [15], anxiety [16], and depression [16,17].

Annual data from the Pew Research Center on Internet, Science & Tech provide the only national benchmarks of the uptake and prevalence of social media and technology use among US teenagers and adults. These reports frequently present a breakdown of the demographic characteristics associated with use of digital technologies, including by age. However, there is limited detail on the characteristics of technology users within a specific age group, except for teens. Young adults have been characterized as hard-to-reach with regard to health promotion and disease prevention, but are widely accessing social media. Given young adults’ unique relationship with technology [18], social media could be a powerful venue for health intervention delivery, including mental health and substance use interventions, in this group. The purpose of this study was to provide updated estimates of social media use from 2014 to 2016, and correlates of social media use, including psychosocial measures, common risk behaviors, and access to digital technology in data collected from a national sample of US young adults aged 18-24 years in 2016. The broader goal of this work is to disseminate these data to inform health intervention efforts for young adults.

## Methods

### Study Sample

The Truth Initiative Young Adult Cohort Study was designed to understand the trajectories of tobacco use in a young adult population using a longitudinal cohort sample. Details of the cohort have been described elsewhere [19]. Briefly, the cohort comprises a nationally representative sample of young adults of ages 18-34 years drawn from GfK (*Gesellschaft für Konsumforschung* [Society for Consumer Research])’s KnowledgePanel. KnowledgePanel is a Web-based panel of adults of ages 18 years and older that covers those who do and do not use the Internet in the United States [20]. The panel was recruited via address-based sampling, a probability-based random sampling method that provides a statistically valid representation of the US population, including cell phone-only households; at the time of the baseline survey, the KnowledgePanel was one of the only providers that used this methodology. African American and Hispanic young adults were oversampled to ensure sufficient sample sizes for subgroup analyses. The validity of this methodology has been reported previously [21,22], and it has been used broadly in the peer-reviewed medical literature [23-27]. The baseline survey (Wave 1; n=4201) was conducted in July 2011, with subsequent assessments occurring approximately every six months; the study is ongoing with the most recent Wave 10 collected in October 2016. The cohort is refreshed at each wave to maintain the initial sample size.

This study used data from responses of participants aged 18-24 years to the Wave 7 survey (October 2014, N=1259) and the Wave 9 survey (February 2016, N=1023), as these two waves included the relevant items on social media use. In Wave 9, 34 participants (3.3%, 34/1023) were missing data on covariates and excluded, yielding an analytic sample of N=989. For the full sample of respondents of ages 18-34 years, the panel recruitment rate was 13.9% in Wave 7 and 13.2% in Wave 9 [28]. In 64% of the identified households, one member completed a core profile survey in which the key demographic information was collected (profile rate—PROR). For this particular study, only one panel member per household was selected at random to be part of the study sample and no members outside the panel were recruited. The response rate (RR6) for Wave 7 was 61.1% and 60.7% for Wave 9. Thus, the cumulative response rate (CUMRR1; the product of these three rates) was 5.4% for Wave 7 and 5.1% for Wave 9. This study was approved by the Chesapeake Institutional Review Board, Inc, and consent was collected from participants before survey self-administration.

### Measures

#### Social Media Site Use

Participants were asked about frequency of use for 10 social media sites: Twitter, Facebook, Instagram, Snapchat, Vine, Pinterest, Tumblr, Google+, LinkedIn, YouTube, and other sites not otherwise specified. Response options were “never,” “less than 1 time per month,” “monthly,” “weekly,” “daily,” or “multiple times per day.” Participants who reported that they used a social media site at least weekly were defined as regular users of that site. Participants who reported regular use of at least one social media site were classified as regular social media users. In 2014, participants were also asked to enumerate, in an open-ended response, approximate counts of their Facebook friends, Twitter followers, people they follow on Twitter, Instagram followers, and people they follow on Instagram.

#### Access to Digital Technology

All participants were asked about their access to digital devices, using the following item: “Which of the following types of digital devices do you have access to or use? Select all that apply” with the following response choices: “A smartphone with Internet access (for example, iPhone, Android, Blackberry etc)”; “A tablet with Internet access (for example, iPad, Samsung Galaxy Tab etc)”; “A desktop or laptop computer with Internet access”; “A Smart TV or video game console that has Internet access”; “A cell phone without Internet access (to talk and text).” Participants could also endorse the single response choice, “None of these.”

#### Other Covariates

As part of KnowledgePanel routine baseline data collection, participants provided information on age at study entry, gender, race/ethnicity (white, non-Hispanic; black, non-Hispanic; other, non-Hispanic; and Hispanic), and highest education completed (less than high school, high school, and some college or greater). GfK conducted hot deck imputation to handle missing data on age, gender, race/ethnicity, and education level. A measure of subjective financial status, developed and validated in the Truth Initiative Young Adult Cohort Study [29], was included as it has been shown to be a more robust measure of socioeconomic status for young adults than income or educational attainment. The item asked: “Considering your own income and the income from any other people who help you, how would you describe your overall personal financial situation?” with response options “don’t meet basic needs,” “just meet basic needs,” “meet needs with a little left,” and “live comfortably.” Two separate variables to assess for symptoms consistent with current depression and current anxiety were created from the two-item Patient Health Questionnaire (PHQ-2) [30] or the two-item Generalized Anxiety Disorder (GAD) scale [31], respectively. Using a four-point scale (0 = “not at all” and 3 = “nearly every day”), the two-item PHQ queries about the frequency of depressed mood and loss of interest in pleasant activities, whereas the two-item GAD queries about the frequency of uncontrollable worry and feelings of anxiety in the past two weeks. Per the scoring rubric for each scale, individuals who received a score at or above the cut-off (>3) were coded as having symptoms of depression or anxiety, respectively. Past 30-day alcohol and marijuana use were determined by two items. For alcohol, the first item asked about frequency of drinking alcohol in the past year (“never,” “monthly or less,” “2-4 times per month,” “2-3 times per week,” “4 or more times per week”); for marijuana use, participants were asked about the frequency of their current use, with response options “every day,” “some days,” and “not at all.” Those who reported any use of alcohol or marijuana were then queried about the frequency of use in the past 30 days, with respondents who reported using ≥1 day in the past month defined as current users.

### Analytic Plan

All analyses were performed using Stata/SE 14.0 (StataCorp, 2014) in August 2016. Poststratification weights were used to offset any nonresponse or noncoverage bias and produce nationally representative estimates specific to each wave of data collection. Missing data were handled with list-wise deletion per Stata’s survey procedures as there was a low proportion of missing data on relevant covariates.

Bivariate analyses were conducted using the survey commands in Stata to provide the estimates of use of each social media site and overlap between use of sites, and to assess the correlates of using a greater number of social media sites regularly. Differences in prevalence estimates were assessed using *P* values from the crude log-binomial regressions of regular use of each social media site and crude linear regressions of number of social media sites on demographics (Wald tests, *P*<.05). Adjusted log-binomial regressions were used to examine the associations of sociodemographic variables on regular use of each social media site and on the number of social media sites regularly used (*P*<.05). Analyses conducted for the items on access to digital technology comprised univariate and bivariate analyses examining the overall prevalence of access to specific digital devices and differences in use of various devices by sociodemographic characteristics.

## Results

### Participant Characteristics

In 2014, 89.42% (1126/1298) of young adults aged 18-24 years reported regular use of at least one social media site (Table 1). This increased to 97.5% (965/989) of young adults in 2016. In 2016, there were 989 (weighted) young adults aged 18-24 years who were regular social media users (Table 2). Of these, 49.7% (492/989) were male, 52.3% (517/989) white, non-Hispanic, 58.1% (575/989) had at least some college education, and 63.4% (628/989) reported a financial situation that at least met their needs with a little left. Also, 6.0% (60/989) of the sample reported symptoms of depression and 7.0% (70/989) reported symptoms of anxiety.

**Table 1:** Percentage of US young adults aged 18-24 years who regularly use social media sites.

Social media sites	Percentage among all young adults (18-24 years of age)				Percentage among young adults who regularly^a^ use social media			
	Wave 7; N=1259	Wave 9; N=989	Relative percent difference compared with Wave 7	Absolute percent difference compared with Wave 7	Wave 7; N=1126; 89.44%	Wave 9; N=965; 97.57%	Relative percent difference compared with Wave 7	Absolute percent difference compared with Wave 7
Facebook	947 (75.24)	758 (76.6)	102	1	947 (84.15)	758 (78.5)	93	−6
YouTube	814 (64.68)	541 (54.6)	84	−10	814 (72.33)	541 (56.0)	77	−16
Instagram	465 (36.89)	779 (78.7)	213	42	465 (41.26)	779 (80.7)	196	39
Snapchat	387 (30.75)	789 (79.7)	259	49	387 (34.39)	789 (81.7)	238	47
Twitter	347 (27.58)	754 (76.2)	276	49	347 (30.85)	754 (78.1)	253	47	
Google+	278 (22.06)	757 (76.5)	347	54	278 (24.67)	757 (78.5)	318	54
Pinterest	220 (17.51)	700 (70.8)	404	53	220 (19.58)	700 (72.6)	371	53
Tumblr	140 (11.08)	825 (83.4)	752	72	140 (12.39)	825 (85.5)	690	73
Vine	108 (8.56)	818 (82.7)	966	74	108 (9.57)	818 (84.8)	885	75
LinkedIn	106 (8.44)	761 (76.9)	911	68	106 (9.44)	761 (78.9)	836	69
Other sites	49 (3.86)	808 (81.7)	2119	78	49 (4.31)	808 (83.7)	1942	79

^a^Regular use is defined as using a site multiple times a day, daily, or weekly.

### Social Media Use

Table 1 compares the popularity of 10 social media sites available in 2014 and 2016. Among regular social media users (weighted n=1126) in 2014, the top 5 social media sites were Facebook (947/1126, 84.15%), YouTube (814/1126, 72.33%), Instagram (465/1126, 41.26%), Snapchat (387/1126, 34.39%), and Twitter (347/1126, 30.85%). Among regular social media users in 2016 (weighted n=965) the top 5 sites were Tumblr (825/965, 85.5%), Vine (818/965, 84.8%), Snapchat (789/965, 81.7%), Instagram (779/965, 80.7%), and LinkedIn (761/965, 78.9%). Google+ (757/965, 78.5%), Facebook (758/965, 78.5%), and Twitter (754/965, 78.1%) had similar levels of regular use.

In 2014, respondents listed the greatest number of friends/followers on Facebook (n=292), followed by Instagram (followers: n=167; people you follow on Instagram: n=160) and Twitter (followers: n=111; people you follow on Twitter: n=128).

Correlates of regular use among the top 5 social media sites in 2016 are presented in Table 2. Compared with males, females were slightly less likely to regularly use Tumblr (n=426 vs 399; 86.6% vs 80.2%). There were no significant differences in regular use of a site by race/ethnicity or education, with the exception of LinkedIn. A greater proportion of regular LinkedIn users were black, non-Hispanic (PR 1.12, 95% CI 1.02-1.23) than white, non-Hispanic. Prevalence of LinkedIn use was also higher among those with less than high school education (PR 1.25, 95% CI 1.12-1.39) or a high school education (PR 1.24, 95% CI 1.15-1.33) compared with those with some college education. Respondents who reported subjective financial situation as “just meeting basic needs” were less likely to report regular use of Tumblr compared with those who “meet needs with a little left” (PR 0.91, 95% CI 0.85-0.98). Young adults who “don’t meet basic needs” had a significantly higher prevalence of using Snapchat regularly compared with those who “meet needs with a little left” (PR 1.12, 95% CI 1.02-1.24). There were no differences in regular use of any of the top 5 most popular social media sites by mental health covariates, but past 30-day alcohol users reported a lower prevalence of Snapchat use (PR 0.88, 95% CI 0.82-0.94).

Respondents reported regularly using an average of 7.6 social media sites (range 0-11 sites), with 3.0% (30/989) reporting regular use of 0-1 social media sites, 2.8% (27/989) using 2 or 3 sites, 9.1% (90/989) using 4 or 5 sites, and 85.1% (843/989) using 6 or more sites regularly. Table 3 presents the mean number of social media sites used regularly and correlates of the number of sites used regularly. In bivariate and multivariable analyses, having a high school education and past 30-day alcohol use were positively associated with using a greater number of social media sites compared with those with some college education and no past 30-day alcohol use, respectively.

**Table 2:** Percentages and correlates of regular social media use, by site, among US young adults aged 18-24 years (weighted n=989).

Variable		Total	Tumblr		Vine		Snapchat		Instagram		LinkedIn	
		(N^a^=989)	(N^a^=825)		(N^a^=818)		(N^a^=789)		(N^a^=779)		(N^a^=761)	
		%	%	PR^b^ (95% CI)	%	PR (95% CI)	%	PR (95% CI)	%	PR (95% CI)	%	PR (95% CI)
Overall			83.4		82.7		79.7		78.7		76.9	
**Sex**												
	Male	49.8	86.6	Ref^c^	84.2	Ref	77.7	Ref	78.7	Ref	78.9	Ref
	Female	50.3	80.2	0.93 (0.87-0.98)^d^	81.2	0.96 (0.91-1.03)	81.8	1.05 (0.98-1.13)	78.7	1.00 (0.93-1.08)	75.0	0.95 (0.88-1.02)
**Race/ethnicity**												
	White, NH^e^	52.3	84.3	Ref	81.0	Ref	79.9	Ref	79.9	Ref	75.3	Ref
	Black, NH	12.9	87.2	1.03 (0.95-1.13)	84.6	1.04 (0.94-1.16)	79.3	0.99 (0.88-1.11)	79.9	1.00 (0.89-1.12)	84.6	1.12 (1.02-1.23)^d^
	Other, NH	8.8	83.6	0.99 (0.89-1.11)	82.9	1.02 (0.90-1.17)	77.9	0.97 (0.84-1.13)	76.8	0.96 (0.82-1.13)	70.4	0.93 (0.77-1.13)
	Hispanic	26.0	79.6	0.94 (0.87-1.02)	85.0	1.05 (0.98-1.13)	80.2	1.00 (0.92-1.09)	76.3	0.96 (0.87-1.05)	78.6	1.04 (0.96-1.14)
**Education**												
	Less than high school	12.1	83.1	1.00 (0.89-1.12)	86.0	1.06 (0.95-1.18)	85.1	1.09 (0.97-1.22)	80.5	1.03 (0.91-1.16)	87.5	1.25 (1.12-1.39)^d^
	High school	29.8	84.4	1.02 (0.95-1.09)	84.0	1.03 (0.96-1.11)	80.6	1.03 (0.95-1.12)	78.5	1.00 (0.92-1.09)	86.4	1.24 (1.15-1.33)^d^
	Some college	58.1	82.9	Ref	81.3	Ref	78.2	Ref	78.4	Ref	69.9	Ref
**Financial Situation**												
	Don't meet basic needs	6.6	80.0	0.91 (0.79-1.05)	88.0	1.07 (0.96-1.18)	90.6	1.12 (1.02-1.24)^d^	77.2	0.96 (0.81-1.13)	80.6	1.03 (0.88-1.20)
	Just meet basic needs	30.0	80.1	0.91 (0.85-0.98)^d^	83.0	1.00 (0.93-1.08)	75.9	0.94 (0.86-1.03)	74.5	0.92 (0.84-1.01)	76.1	0.97 (0.89-1.06)
	Meet needs with a little left	38.0	87.9	Ref	82.6	Ref	80.6	Ref	80.6	Ref	78.5	Ref
	Live comfortably	25.5	81.5	0.93 (0.86-1.00)	81.0	0.98 (0.90-1.06)	80.1	0.99 (0.91-1.08)	81.3	1.01 (0.93-1.10)	74.5	0.95 (0.87-1.04)
**Depression**												
	No	94.0	83.6	Ref	82.7	Ref	79.5	Ref	79.0	Ref	76.5	Ref
	Yes	6.0	79.2	0.95 (0.82-1.09)	82.1	0.99 (0.87-1.13)	82.6	1.04 (0.91-1.18)	73.7	0.93 (0.78-1.11)	82.8	1.08 (0.96-1.22)
**Anxiety**												
	No	93.0	83.4	Ref	83.5	Ref	79.7	Ref	79.0	Ref	76.9	Ref
	Yes	7.0	82.8	0.99 (0.88-1.12)	71.9	0.86 (0.73-1.01)	80.5	1.01 (0.89-1.15)	74.9	0.95 (0.81-1.11)	77.8	1.01 (0.88-1.16)
**Past 30-day alcohol use**												
	No	53.0	83.2	Ref	85.0	Ref	84.5	Ref	81.2	Ref	79.8	Ref
	Yes	47.0	83.6	1.00 (0.95-1.07)	80.1	0.94 (0.89-1.00)	74.3	0.88 (0.82-0.94)^b^	75.9	0.93 (0.87-1.00)	73.7	0.92 (0.86-0.99)^d^
**Past 30-day marijuana use**												
	No	88.0	83.2	Ref	82.8	Ref	80.0	Ref	79.0	Ref	77.1	Ref
	Yes	12.0	85.0	1.02 (0.93-1.12)	81.4	0.98 (0.89-1.08)	77.9	0.97 (0.87-1.09)	76.3	0.96 (0.85-1.09)	75.3	0.98 (0.87-1.10)

^a^Weighted.

^b^PR: prevalence ratio.

^c^Ref: Reference category.

^d^Denote statistical significance at *P*<.05.

^e^NH: non-Hispanic.

Table 4 presents a matrix of the overlap between use of the top 5 social media sites among young adults. These findings highlight significant overlap (81%-90%) in use of multiple social media sites.

### Access to Digital Technology

Access to specific types of digital technologies among young adults was as follows: a smartphone with Internet access, 86.9% (860/989); a desktop or laptop computer with Internet access, 74.3% (736/989); a tablet with Internet access, 40.6% (401/989); a smart TV or video game console with Internet access, 29.0% (287/989); a cell phone without Internet access, 11.5% (114/989); none of these, 3.0% (30/989; Table 5). Females were significantly less likely to report having a smart TV or video game console with Internet access compared with males (PR 0.76, 95% CI 0.62-0.94). Females, however, reported greater access to a smartphone with Internet access (PR 1.07, 95% CI 1.01-1.14) compared with males. Compared with non-Hispanic whites, non-Hispanic black young adults had a significantly lower prevalence of access to a smartphone with Internet access (77.4%, 99/128 vs 89.6%, 463/517; PR 0.86, 95% CI 0.75-0.99), a desktop or laptop with Internet access (59.6%, 76/128 vs 80.4%, 416/517; PR 0.74, 95% CI 0.60-0.91), and a smart TV or video game console with Internet access (16.2%, 21/128 vs 30.2%, 156/517; PR 0.54, 95% CI 0.32-0.89). Hispanic young adults also reported lower prevalence of access to a desktop or laptop with Internet access (68.6%, 176/257 vs 80.4%, 416/517; PR 0.85, 95% CI 0.78-0.96). Respondents with a high school education or less reported significantly lower prevalence of access to a smartphone (80.3%, 333/414 vs 91.6%, 527/575; PR 0.88, 95% CI 0.82-0.94), a tablet (33.6%, 139/414 vs 45.6%, 262/575; PR 0.74, 95% CI 0.61-0.89), and a desktop or laptop with Internet (65.0%, 269/414 vs 81.1%, 466/575; PR 0.80, 95% CI 0.72-0.89) compared with those with at least some college education. Past 30-day alcohol users reported a higher prevalence of access to a smartphone with Internet (PR 1.12), a desktop or laptop with Internet (PR 1.09), and a smart TV or video game console with Internet (PR 1.31) compared with those who did not report past 30-day alcohol use. Past 30-day marijuana users reported a significantly higher prevalence of access to a smart TV or video game console with Internet access (PR 1.52) compared with nonusers.

**Table 3:** Correlates of number of social media sites regularly used (defined as using a site multiple times a day, daily, or weekly) among US young adults aged 18-24 years (weighted n=989).

Variable		Mean (SE)^a^	LR^b^	95% CI	aLR^b^	95% CI
**Sex**						
	Male	7.74 (0.13)	Ref		Ref	
	Female	7.38 (0.10)	−0.05	−0.09 to 0.00	−0.04	−0.09 to 0.00	
**Race**						
	White, non-Hispanic	7.53 (0.10)	Ref		Ref	
	Black, non-Hispanic	7.80 (0.26)	0.04	−0.03 to 0.11	0.02	−0.05 to 0.09
	Other, non-Hispanic	7.37 (0.32)	−0.02	−0.11 to 0.07	−0.04	−0.13 to 0.05	
	Hispanic	7.57 (0.18)	0.01	−0.05 to 0.06	0.00	−0.06 to 0.05
**Education**						
	Less than high school	7.99 (0.34)	0.08	0.00-0.17	0.07	−0.01 to 0.16
	High school	7.80 (0.16)	0.06	0.01-0.11^c^	0.05	0.00-0.10^c^
	Some college	7.35 (0.09)	Ref		Ref	
**Financial situation**						
	Don't meet basic needs	7.83 (0.26)	0.03	−0.04 to 0.10	0.01	−0.06 to 0.08
	Just meet basic needs	7.40 (0.17)	−0.03	−0.08 to 0.02	−0.04	−0.09 to 0.02
	Meet needs with a little left	7.62 (0.12)	Ref		Ref	
	Live comfortably	7.59 (0.17)	0.00	−0.06 to 0.05	−0.01	−0.06 to 0.04
**Depression**						
	No	7.57 (0.09)	Ref		Ref	
	Yes	7.41 (0.33)	−0.02	−0.11 to 0.07	−0.02	−0.16 to 0.12
**Anxiety**						
	No	7.57 (0.08)	Ref		Ref	
	Yes	7.38 (0.38)	−0.03	−0.13 to 0.08	−0.02	−0.17 to 0.13	
**Past 30-day alcohol use**						
	No	7.80 (0.11)	Ref		Ref	
	Yes	7.30 (0.12)	−0.07	−0.11 to −0.02^c^	−0.06	−0.10 to −0.01^c^
**Past 30-day marijuana use**						
	No	7.57 (0.09)	Ref		Ref	
	Yes	7.48 (0.27)	−0.01	−0.09 to 0.06	0.00	−0.07 to 0.08	

^a^SE: standard error.

^b^Crude (LR) and adjusted linear regressions (aLR) with significance at *P*<.05.

^c^Denote statistical significance at *P*<.05.

**Table 4:** Use of multiple social media sites among regular (defined as using a site multiple times a day, daily, or weekly) users (US young adults aged 18-24 years, weighted n=989). The table presents the % of regular users (ages 18-24 years) of each particular site who use another particular site (eg, 89% of regular users of Tumblr also regularly use Vine).

Other social media use	Use Tumblr, n (%)	Use Vine, n (%)	Use Snapchat, n (%)	Use Instagram, n (%)	Use LinkedIn, n (%)
% of Tumblr users (n=825) who…	-	734 (89)	691 (84)	679 (82)	678 (82)
% of Vine users (n=818) who…	734 (90)	-	697 (85)	687 (84)	666 (81)
% of Snapchat users (n=789) who…	691 (88)	697 (88)	-	684 (87)	647 (82)
% of Instagram users (n=779) who…	679 (87)	687 (88)	684 (88)	-	631 (81)
% of LinkedIn users (n=761) who…	678 (89)	666 (88)	647 (85)	631 (83)	-

**Table 5:** Correlates of access to specific types of digital technologies among US young adults aged 18-24 years (weighted n=989).

Participant characteristics		A smartphone with Internet access (87%)	A tablet with Internet access (41%)	A desktop or laptop with Internet access (74%)	A smart TV or video game console with Internet access (29%)	A cell phone without Internet access (11%)
		PR^a^ (95% CI)	PR (95% CI)	PR (95% CI)	PR (95% CI)	PR (95% CI)
**Sex**						
	Male	Ref^b^	Ref	Ref	Ref	Ref
	Female	1.07 (1.01-1.14)^c^	1.11 (0.94-1.31)	0.98 (0.90-1.07)	0.76 (0.62-0.94)^c^	0.67 (0.45-1.00)
**Race**						
	White, NH^d^	Ref	Ref	Ref	Ref	Ref
	Black, NH	0.86 (0.75-0.99)^c^	0.74 (0.51-1.07)	0.74 (0.60-0.91)^c^	0.54 (0.32-0.89)^c^	1.57 (0.86-2.85)
	Other, NH	0.96 (0.84-1.09)	1.19 (0.88-1.60)	0.96 (0.82-1.12)	0.60 (0.34-1.08)	0.37 (0.12-1.11)
	Hispanic	0.97 (0.90-1.03)	1.07 (0.89-1.28)	0.85 (0.77-0.95)^c^	1.21 (0.97-1.51)	1.02 (0.63-1.64)
**Education**						
	Less than high school	0.81 (0.69-0.94)^c^	0.65 (0.43-0.97)^c^	0.65 (0.52-0.83)^c^	1.01 (0.68-1.51)	1.47 (0.73-2.94)
	High school	0.91 (0.84-0.98)^c^	0.77 (0.63-0.94)^c^	0.86 (0.78-0.96)^c^	1.04 (0.81-1.33)	1.44 (0.93-2.25)
	Some college	Ref	Ref	Ref	Ref	Ref
**Financial Situation**						
	Don’t meet basic expenses	0.74 (0.60-0.92)^c^	0.67 (0.44-1.02)	0.71 (0.54-0.93)^c^	0.42 (0.20-0.88)^c^	1.27 (0.60-2.67)
	Just meet basic expenses	0.87 (0.80-0.94)^c^	0.65 (0.52-0.82)^c^	0.90 (0.80-1.00)	0.84 (0.63-1.10)	1.38 (0.81-2.35)
	Meet needs with a little left	Ref	Ref	Ref	Ref	Ref
	Live comfortably	1.00 (0.94-1.06)	1.12 (0.93-1.34)	1.05 (0.96-1.16)	1.11 (0.87-1.42)	1.22 (0.72-2.07)
**Depression**						
	No	Ref	Ref	Ref	Ref	Ref
	Yes	0.97 (0.84-1.11)	0.91 (0.63-1.32)	0.87 (0.71-1.08)	0.90 (0.55-1.47)	1.74 (0.94-3.21)
**Anxiety**						
	No	Ref	Ref	Ref	Ref	Ref
	Yes	0.97 (0.85-1.11)	1.04 (0.76-1.41)	0.89 (0.73-1.08)	0.97 (0.64-1.47)	1.41 (0.75-2.63)
**Past 30-day alcohol use**						
	No	Ref	Ref	Ref	Ref	Ref
	Yes	1.12 (1.05-1.19)^c^	1.01 (0.86-1.19)	1.09 (1.00-1.19)^c^	1.31 (1.05-1.62)^c^	0.78 (0.51-1.20)
**Past 30-day marijuana use**						
	No	Ref	Ref	Ref	Ref	Ref
	Yes	1.02 (0.93-1.12)	0.88 (0.67-1.15)	0.97 (0.85-1.11)	1.52 (1.17-1.98)^c^	0.81 (0.45-1.47)

^a^PR: prevalence ratio.

^b^Ref: Reference category.

^c^Denote statistical significance at *P*<.05.

^d^NH: non-Hispanic.

Access to all Internet-enabled devices varied by subjective financial situation was as follows. Those who reported meeting needs comfortably or meeting needs with a little left reported greater access to a smartphone, a tablet with Internet access, a desktop or laptop with Internet access, and a smart TV or video game console with Internet access than those who “don’t meet” or “just meet” basic expenses (*P*<.05). Access to a cell phone without Internet access was low overall (11.5%, 114/989) and did not vary by subjective financial situation. Those who “don’t meet basic expenses” reported lower prevalence of access to a smartphone, a desktop or laptop, and a smart TV or video game console with Internet, and those who “just meet basic expenses” reported lower prevalence of access to a smartphone and tablet with Internet access, compared with those who “meet needs with a little left.” In the group at greatest socioeconomic disadvantage (“don’t meet basic needs”) (n=65), 68.4% (n=45) reported access to a smartphone, 30.0% (n=20) to a tablet, 54.7% (n=36) to a desktop or laptop, 13.0% (n=8) to a smart TV or video game console with Internet access, and 12.3% (n=8) to a cell phone without Internet access. Compared with those who “don’t meet basic needs,” those who “just meet basic needs” (n=297) had similar levels of access to a smartphone (79.8%, n=237), tablet (29.4%, n=87), desktop or laptop (69.2%, n=205), smart TV or video game console (25.8%, n=76), and cell phone without Internet (13.4%, n=40).

## Discussion

### Principal Findings

According to Pew Research, 99% of US young adults aged 18-29 years in 2016 used the Internet [32] and 90% of these young adults used a social networking site as of 2015 [4]. As of September 2014, 87% of Internet-using young adults reported using Facebook, 53% reported using Instagram, 37% reported using Twitter, and 34% reported using Pinterest [33]. This Pew Research survey did not report on YouTube use. Our study shows that social media use has continued to increase and regular social media use was nearly ubiquitous in 2016 in a national sample of young adults aged 18-24 years. Additionally, young adults surveyed in 2016 reported regular use of different sites than in 2014 (ie, Tumblr, Vine, Snapchat, Instagram, and LinkedIn). The fact that most sites allow for multimedia content reflect the rising importance of visual content in Internet communication [34], particularly among young people. Lower use of YouTube in 2016 suggests that young adults may be accessing YouTube video content secondarily via other social media sites (eg, links in Twitter) and attributing the content to the primary site used. Participants used a high mean number of social media sites regularly and there was substantial overlap in use of social media sites.

In addition to providing updated estimates of social media use, this study found that while there were no consistent correlates of use of particular social media sites, there were a few relationships that deserve further exploration. First, there was a higher prevalence of LinkedIn use by black young adults and those with less than a college education. LinkedIn is an employment-oriented social media site that offers professional networking opportunities; higher LinkedIn use among young adults with lower education may reflect job-seeking among young people not enrolled in college. Given the professional focus of this site, it is unlikely to yield much information about the health behaviors of young people. Two other sites may provide more insight into the depiction of health behaviors on the Internet: Snapchat and Tumblr. Our study found there was a higher prevalence of Snapchat use among young adults reporting the greatest socioeconomic disadvantage. There was also a lower prevalence of Tumblr use among females and those who “just meet basic needs.” Tumblr, a social blogging platform, allows users to share and discuss multimedia content (text, photos, quotes, links, music, videos) and customize their blog using embedded tools. Approximately 32% of Tumblr bloggers are 18-24 years of age, and 67% are under age 35 [35]. Tumblr users can remain anonymous, which facilitates a relatively high degree of disclosure and sharing, particularly around sensitive topics [36-38]. Snapchat enables users to share photos and short videos with closed networks of friends or to broader unknown networks that disappear in 24 hours or less. It features tools for customizing photos/videos with filters, stickers, and drawings. High use of these sites reflects the potential importance of anonymity, creativity, and ephemerality to social media users. Emerging research examines how people use these sites to portray their engagement in health-risk behaviors (eg, sex, alcohol use, tobacco use) [39-48]. In line with social cognitive theory [9], we would expect social observation and interaction via social media to influence beliefs about health-risk behaviors or the behaviors themselves. To date, limited research exists on young adult exposure to risk behaviors via social media and the impact of such exposure on subsequent risk behavior [49-54].

Mobile devices are a primary channel for social media: Pew data indicate that in 2015, 85% of young adults aged 18-29 years had a smartphone and 91% of these individuals use a social networking site on the phone [55]. Our findings are consistent, showing that in 2016, 86.9% (860/989) of young adults aged 18-24 years had a smartphone. Novel findings from our study, however, highlight disparities in access to digital technologies among US young adults by race/ethnicity, education, and subjective financial status. Black young adults, those with less than a college education, and those who “don’t meet” or “just meet” basic expenses were significantly less likely to have access to a smartphone with Internet access compared with whites, those with a college education, and those who “meet needs with a little left,” respectively. A similar pattern emerged regarding access to a desktop or laptop with Internet access. Interestingly, this did not result in a greater proportion of these respondents reporting access to a cell phone without Internet. Smartphone access remained relatively high, despite these differences, with the lowest prevalence seen in those reporting that they “don’t meet basic needs” (68.4%). Disparities in ongoing social media and Internet access may have important implications for Web-based health interventions seeking to target groups that may be at highest risk.

### Limitations

Limitations of this study include the self-reported nature of social media use and the social media sites identified in the survey. We may have missed other popular social media sites and data on some of the sites used may be erroneous; for example, the high prevalence of use of Google+ likely reflects use of Google’s search engine given the failure of the Google+ social networking site [56]. Similarly, our question regarding access to and use of digital technology did not ask about device ownership as in the Pew studies. Deviations from the Pew Center data may reflect differences in the timing of the surveys, items used, sample-specific differences in survey measurement, including differences in sample sizes, and different sampling and weighting strategies. The study sample’s completion rates and cumulative response rates are similar to that of other health studies that have relied on KnowledgePanel [24-26,57]. The internal validity of our results is not compromised by the panel’s cumulative response rate, and other work suggests that surveys with a low response rate can still be representative of the sample population, even though the risk of nonresponse bias is higher [58,59]. Studies assessing nonresponse to panel recruitment in KnowledgePanel have found little indication of nonresponse bias on core demographic and socioeconomic variables [60,61], and previous estimates from this cohort for key outcomes of interest, such as ever and current cigarette use, are consistent with national survey data [19]. Although missing data were relatively low for our covariates of interest (3.3%), our analytic approach that used list-wise deletion may have introduced bias to our results if missingness is not random.

### Conclusions

There are several mechanisms through which social media interventions can influence health behavior; however, few studies to date have used social media to facilitate health behavior change in young adults [62-68]. Intervention and user-generated content in social media can be a powerful source of influence through peer modeling [69]. Additionally, content delivered via social media may correct misperceptions, offer resources to assist behavior change, and provide opportunities to recruit peer support for behavior change via one’s existing social networks. The findings from the current study may guide the development and implementation of future health interventions for young adults delivered via the Internet or social media sites. Our study highlights that young people are using multiple social media sites regularly and that these sites may provide an accessible venue for delivering health messaging. These messages will need to be tailored for the top social media sites used, including creative use of images, videos, hashtags, and other content to be relevant to the target audience. Intervention exposure in the target population may be maximized through coordinated dissemination of health messages across multiple social media sites. Lower access to Internet-enabled mobile devices among black young adults and socioeconomically disadvantaged young adults does not discount the utility of Web-based health behavior interventions in this group, but highlights that other channels may be needed to complement a Web-based approach in these subgroups. This research also highlights that the top social media sites change rapidly and any social media intervention approach for young adults must be flexible and nimble enough to adapt to new sites, new patterns of use, and new ways of delivering content via social media.

## Abbreviations

GAD: generalized anxiety disorder
GfK: Gesellschaft für Konsumforschung (Society for Consumer Research)
PHQ: patient health questionnaire
SE: standard error
